# Temporal Lobe Epilepsy and Surgery Selectively Alter the Dorsal, Not the Ventral, Default-Mode Network

**DOI:** 10.3389/fneur.2014.00023

**Published:** 2014-03-10

**Authors:** Gaelle Eve Doucet, Christopher Skidmore, James Evans, Ashwini Sharan, Michael R. Sperling, Dorian Pustina, Joseph I. Tracy

**Affiliations:** ^1^Department of Neurology, Thomas Jefferson University, Philadelphia, PA, USA; ^2^Department of Neurosurgery, Thomas Jefferson University, Philadelphia, PA, USA

**Keywords:** default-mode network, dorsal and ventral subdivisions, temporal lobe epilepsy, anterior temporal lobectomy, resting-state, fMRI

## Abstract

The default-mode network (DMN) is a major resting-state network. It can be divided in two distinct networks: one is composed of dorsal and anterior regions [referred to as the dorsal DMN (dDMN)], while the other involves the more posterior regions [referred to as the ventral DMN (vDMN)]. To date, no studies have investigated the potentially distinct impact of temporal lobe epilepsy (TLE) on these networks. In this context, we explored the effect of TLE and anterior temporal lobectomy (ATL) on the dDMN and vDMN. We utilized two resting-state fMRI sessions from left, right TLE patients (pre-/post-surgery) and normal controls (sessions 1/2). Using independent component analysis, we identified the two networks. We then evaluated for differences in spatial extent for each network between the groups, and across the scanning sessions. The results revealed that, pre-surgery, the dDMN showed larger differences between the three groups than the vDMN, and more particularly between right and left TLE than between the TLE patients and controls. In terms of change post-surgery, in both TLE groups, the dDMN also demonstrated larger changes than the vDMN. For the vDMN, the only changes involved the resected temporal lobe for each ATL group. For the dDMN, the left ATL group showed post-surgical increases in several regions outside the ictal temporal lobe. In contrast, the right ATL group displayed a large reduction in the frontal cortex. The results highlight that the two DMNs are not impacted by TLE and ATL in an equivalent fashion. Importantly, the dDMN was the more affected, with right ATL having a more deleterious effects than left ATL. We are the first to highlight that the dDMN more strongly bears the negative impact of TLE than the vDMN, suggesting there is an interaction between the side of pathology and DM sub-network activity. Our findings have implications for understanding the impact TLE and subsequent ATL on the functions implemented by the distinct DMNs.

## Introduction

The default-mode network (DMN) has been identified as one of the most robust and consistent resting-state network [see review of Ref. (1, 2)]. While much about its function remains unclear, research has suggested it is engaged in the maintenance of “tonic” or baseline cognitive processing related to self-awareness, episodic memory, or the modulation of internal (mental) versus external tasks. Others have linked it to anticipatory cognitive processes, the strength of cognitive reserve, or consciousness [see review of Ref. (1)]. Most recently, there has been new evidence that this network is also modulated by the nature of the spontaneous thoughts during a conscious resting-state (3, 4). It primarily consists of posterior cingulate cortex (PCC)/precuneus, ventral anterior cingulate cortex (ACC)/mesial prefrontal cortex, angular gyri, lateral temporal cortex, and mesial temporal lobes. However, a growing number of studies consider this network to be comprised of at least two functionally distinct subdivisions (5, 6): one is composed of dorsal and anterior regions [referred to as the dorsal DMN (dDMN)], and appears active when people engage in self-relevant decisions or affectively laden cognitive processes. The second division involves posterior and mesial temporal regions [referred to as the ventral DMN (vDMN)], and engages during decision-making related to constructing a mental scene, particularly a scene called up from episodic or semantic memory.

Investigated as a functional marker for neurological pathologies, several studies have reported that DMN activity is, indeed, altered by neurologic pathologies such as Alzheimer’s disease (7) or schizophrenia (8). Given the role of the DMN in temporal lobe functions such as memory processing and conscious awareness, increasing our understanding of temporal lobe epilepsy (TLE) will require sophisticated analysis of this disorder’s impact on the DMN. With regard to epilepsy, this network has been described as perturbed during both ictal (and subsequently, transitory loss of consciousness) (9) and interictal (10–13) states. To our knowledge, however, only a few studies have specifically investigated the DMN in unilateral TLE patients at rest through fMRI (10–12). Existing studies demonstrate abnormal reduced activity in this network compared to healthy controls, with distinct effects depending on the hemisphere with epileptic pathology. The effects of the standard surgery for intractable epilepsy [e.g., anterior temporal lobectomy (ATL)] on the DMN are still largely unknown. Indeed, to date, only one study has investigated this network and its changes prior to and after epilepsy surgery (10). In this study, McCormick et al. described connectivity changes involving the precuneus post-surgery relative to pre-surgery. These authors, however, did not explore the two specific sub-networks (dDMN and vDMN). Therefore, questions remain as to how the DMN subdivisions may be differentially affected by the resection of the temporal lobe, the laterality of the pathology, and the potential neuroplastic or compensatory responses generated post-surgery. In this sense, ATL provides a valuable model for testing the impact of structural changes in the DMN, as this procedure includes resection of several parts of the network, most notably the lateral and the mesial temporal regions.

In this context, we sought to expand our knowledge about the effect of both TLE and ATL on the DMN’s subdivisions (dDMN and vDMN). We utilize resting-state fMRI data from 29 unilateral TLE patients (13 left, 16 right), who underwent standard en bloc resection of their epileptogenic temporal lobe (standard ATL), and 14 healthy matched controls. Using group independent component analysis (ICA), we identified the ventral and dDMNs. We hypothesized that the spatial extent of each DMN subdivision will differ between left and right TLE patients. More specifically, we expected that the left TLE patients will show more abnormalities than the right TLE, as it has been suggested that left TLE patients generally have more functional impairments at rest than right-sided patients (14, 15). We also hypothesized that ATL surgery will not affect the two networks in the same manner, as it will remove distinct regions in the temporal lobe within each network. We expected the vDMN to show more changes that the dDMN as it is this sub-network that includes the epileptogenic mesial temporal lobe, potentially fostering distinct neuroplasticity mechanisms and compensatory responses. It will increase our understanding of the neuroplasticity responses that one can expect to emerge for each DMN network post-surgery.

## Materials and Methods

### Participants

A total of 14 healthy age-matched controls and 29 patients with refractory unilateral TLE (13 left and 16 right) were recruited from the Thomas Jefferson University Comprehensive Epilepsy Center. All the patients underwent a standard en bloc ATL to remove their epileptogenic temporal lobe. In detail, this surgery included a unilateral ATL and amygdalohippocampectomy [approximately 4–6 cm from the temporal pole with the size smaller for the left (language dominant) temporal lobe patients]. Note that all of our participants, controls and patients, were left hemisphere (LH) dominant as verified by task-fMRI (e.g., verb generation procedure). Details of the Thomas Jefferson Comprehensive Epilepsy Center algorithm for surgical decision-making are described by Sperling et al. (16). A combination of video/surface EEG (at least 96 h), MRI, PET, and neuropsychological testing was used to lateralize the side of seizure focus. All patient participants met the following inclusion criteria: unilateral temporal lobe seizure focus, concordant MRI, and/or PET findings of temporal lobe abnormality with no non-concordant data. TLE patients were excluded from the study for any of the following reasons: previous brain surgery; extra-temporal lesions; medical illness with central nervous system impact other than epilepsy; extra-temporal or multi-focal epilepsy; contraindications to MRI; psychiatric diagnosis other than an axis-I depression or anxiety disorder; or hospitalization for any axis-I disorder listed in the Diagnostic and Statistical Manual of Mental Disorders, IV. Patients provided written informed consent. Table 1 outlines the patients’ demographic and clinical characteristics.

**Table 1:** **Clinical information and characteristics of the patients**.

Pathology	Participants	Gender	Age at the pre-surgery scan date (years)	Age at seizure onset	Seizure type	Temporal pathology^a^	Duration of first scan-surgery (days)	Duration of second scan-surgery (days)	Seizure outcome class
Left TLE	1	M	56.4	16	SPS; rare CPS	Cavernoma	38	915	1
Left TLE	2	F	43.9	3	CPS; rare GTCS	HS	311	251	1
Left TLE	3	F	60.2	53	CPS/SPS	Mild subpial gliosis, no HS	90	1062	1
Left TLE	4	M	35.9	20	CPS	Gliosis and HS	61	1055	1
Left TLE	5	F	41.2	5	CPS/SPS	Gliosis and HS	586	181	5
Left TLE	6	F	42.3	38	CPS	Gliosis and HS	19	244	1
Left TLE	7	F	60.5	13	CPS	Gliosis and HS	34	364	1
Left TLE	8	M	60.0	50	CPS	Gliosis and HS	23	237	1
Left TLE	9	F	25.4	18	CPS with sec GTCS	Low grade glioma	200	238	1
Left TLE	10	F	31.3	14	CPS	Mild gliosis and HS	14	1705	1
Left TLE	11	F	34.7	19	CPS/SPS	Mild subpial gliosis, no HS	42	890	1
Left TLE	12	F	52.2	42	CPS	Gliosis and cavernous angioma	25	1360	1
Left TLE	13	M	34.1	31	CPS/GTCS	Gliosis, no HS	68	208	1
Right TLE	1	F	48.2	16	CPS/SPS	Gliosis and HS	172	787	1
Right TLE	2	F	33.1	2	CPS/rare GTCS	Gliosis and HS	76	641	1
Right TLE	3	F	30.0	10	CPS	Gliosis, no HS	271	530	1
Right TLE	4	F	26.0	21	CPS	Gliosis, cortical dysplasia	36	398	1
Right TLE	5	F	52.4	11	CPS/sec GTCS	Gliosis	64	227	2
Right TLE	6	M	55.3	5	CPS; rare sec GTCS	Gliosis and HS	38	159	1
Right TLE	7	M	57.5	27	CPS	Gliosis, no HS	628	282	2
Right TLE	8	M	28.1	16	CPS	Gliosis	35	255	1
Right TLE	9	M	25.6	19	CPS/SPS	Gliosis	23	245	3
Right TLE	10	M	65.3	20	CPS	Gliosis and HS	36	243	1
Right TLE	11	F	29.7	27	CPS/SPS	Mild gliosis	472	146	4
Right TLE	12	M	39.6	35	CPS	Mild gliosis	101	169	4
Right TLE	13	F	34.5	32	CPS	Gliosis	886	548	1
Right TLE	14	F	47.5	34	CPS	Mild gliosis	109	329	1
Right TLE	15	F	23.4	17	CPS	Gliosis	1451	1391	1
Right TLE	16	M	60.0	51	CPS/GTCS	Gliosis	394	297	2

*F, female; M, male; HS, hippocampal sclerosis; CPS, complex partial seizures; SPS, simple partial seizures; sec GTCS, secondarily generalized tonic/clonic seizures. The seizure outcome classification, from class 1 to 4 is based on the Engel classification (25), class 5 reflects the presence of post-operative pseudo-seizures*.

*^a^ Temporal pathology resulted from the surgical pathology report regarding the resected tissue after the ATL*.

Healthy normal controls (NCs) were recruited from the Thomas Jefferson University community, in order to match the patient participants in age and gender. All controls were free of psychiatric or neurological (central nervous system) disorders based on a health screening measure. This study was approved by the Institutional Review Board for Research with Human Subjects at Thomas Jefferson University and all participants provided a written informed consent.

### MRI data acquisition

All participants underwent magnetic resonance imaging on a 3-T X-series Philips Achieva clinical MRI scanner (Amsterdam, the Netherlands) using an 8-channel head coil. Both the NCs and the TLE patients underwent two identical fMRI scanning sessions. In detail, each patient underwent one pre-surgical (mean = 217 days prior to surgery) and one post-surgical (*m* = 530 days after surgery, minimum of 6 months) scan, while the NCs participated in two fMRI sessions, with parameters identical to the TLE patients at a time interval of at least 6 months. A total of 5 min of a resting-state condition was collected from all participants. Anatomical and functional acquisitions were similar for all patients. A single shot echoplanar gradient echo imaging sequence acquiring T2* signal was used with the following parameters: 120 volumes, 34 axial slices acquired parallel to the AC–PC line, TR = 2.5 s, TE = 35 ms, FOV = 256 mm, 128 × 128 data matrix isotropic voxels, flip angle = 90°, bandwidth = 1.802 (±241.1 kHz). The in-plane resolution was 2 mm × 2 mm and the slice thickness was 4 mm. Prior to collection of the T2* images, T1-weighted images (180 slices) were collected using an MPRage sequence (256 × 256 isotropic voxels; TR = 640 ms, TE = 3.2 ms, FOV = 256 mm, flip angle = 8°) in positions identical to the functional scans to provide an anatomical reference. The in-plane resolution for each T1 slice was 1 mm × 1 mm × 1 mm (axial oblique; angle following the anterior, posterior commissure line). Survey and field reference inhomogeneity images were collected prior to the start of the study. Each EPI imaging series started with three discarded scans to allow for T1 signal stabilization. Subjects lay in a foam pad to comfortably stabilize the head, were instructed to remain still throughout the scan, not fall asleep, and keep their eyes closed during the entirety of the scan.

### Image processing

Data from the patients (pre- and post-surgery scans) and NCs (sessions 1 and 2) were preprocessed identically using SPM8^1^. Slice timing correction was used to adjust for variable acquisition time over slices in a volume, with the middle slice in every volume used as reference. Next, a six-parameter variance cost-function rigid body affine registration was used to realign all images within a session to the first volume. Motion regressors were computed and later used as regressors of no interest. To maximize mutual information, co-registration between functional scans and the MNI305 (Montreal Neurological Institute) template was carried out using six iterations and re-sampled with a seventh-degree B-spline interpolation. Functional images were then normalized and warped into standard space (MNI305) to allow for signal averaging across subjects. We utilized the standard normalization method in SPM8, which minimizes the sum-of-squared differences between the subject’s image and the template (MNI305), while maximizing the prior probability of the transformation. This spatial normalization provided a reliable matching to the MNI template without causing aberrant distortions in the images, both in patients with no brain lesions and those having brain resections and abnormal signal (17). This enabled us to compare brain structures and define the same seed region (see next step) between pre- and post-surgical data. Segmentation of the data in the gray matter, white matter (WM), and cerebro-spinal fluid (CSF) classes was also carried out. All normalized images were smoothed by convolution with a Gaussian kernel, with a full width at half maximum of 8 mm in all directions. For each individual, the time-courses of both WM and CSF were estimated in the relevant brain tissue classes defined at the segmentation step. Sources of spurious variance were then removed from the data through linear regression: six parameters obtained by rigid body correction of head motion and the CSF and WM signals. Finally, fMRI data were temporally filtered using the REST Toolbox (low cutoff frequency = 0.008 Hz – high cutoff frequency = 0.1 Hz) (18, 19). As head motion has been reported to potentially influence resting-state results (20), we utilized *t*-tests to check for differences either across scanning sessions or between our experimental groups. For each individual, we computed the maximum difference (i.e., minimum to maximum) within each of the six realignment parameters computed during preprocessing. No significant differences were observed either across the scanning sessions (pre- to post-) or between the experimental groups (Bonferroni corrected for the six parameters, for an effective alpha of *p* = 0.05).

### Statistical analyses

#### Independent component analysis

Spatial probabilistic ICA (temporal concatenation method) was used to identify resting-state networks. Briefly, preprocessed images from each scan (a total of 86 inputs) were entered into the FMRIB software library (FSL) 4.0.8 Melodic ICA software^2^ (21). This technique performs a spatio-temporal decomposition of the signal without any *a priori* seed, by simultaneously analyzing data from all the subjects (22). We used the Laplace approximation to estimate the number of components. The output resulted in 16 independent components (ICs) common for the entire group of participants. Each IC was associated with a *Z*-map and a time-series. Also, for each component, an effect size value is available for each participant, indicative of the strength of the component in each subject’s data. Each map was thresholded at a posterior probability threshold of *p* = 0.5, using an alternative hypothesis-testing approach based on the fit of a Gaussian/Gamma mixture model (22). In a second step, based on these group ICs, we then applied a dual regression approach to characterize each IC in each subject, through a *Z*-map and an individual time-series (23).

#### Selection of the dorsal and ventral default-mode networks

**Figure 1:**
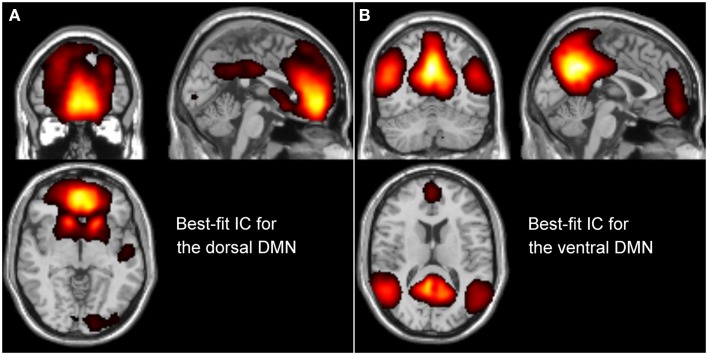
**Description of the best-fit independent components (ICs) for the dorsal DMN (A) and ventral DMN (B), resulted from the group ICA**.

Based on this group decomposition, we identified the two best-fit components for the dDMN and vDMN (Figure 1). For this, we computed the goodness-of-fit (GOF) for each IC, using the dDMN and vDMN templates provided by Shirer et al. (24). In detail, applying Greicius et al.’s method, a linear template-matching procedure was used, which takes the average *z*-score of voxels falling within the template minus the average *z*-score of voxels outside the template (7). These best-fit components reflect the degree to which their best-fit component network matched the default-mode networks template.

The IC selected as the best-fit for the dDMN (Figure 1A) includes a major cluster in the medial prefrontal cortex/ACC, the bilateral caudate nuclei. To a lesser degree, the PCC was also included as well as the right angular gyrus, the left superior temporal cortex, and right calcarine. The IC selected as the best-fit for the vDMN (Figure 1B) includes a large cluster in the medial parietal cortex, including the precuneus, PCC, and retrosplenial cortex. Bilateral angular gyri, the anterior ventral area of the medial prefrontal cortex as well as bilateral parahippocampal gyri (more extensive on the left), bilateral inferior temporal cortices, and bilateral superior/middle frontal cortices were also part of the vDMN.

#### Computation of goodness-of-fit or each individual network

After identifying the two best-fit networks, we computed the GOF for each network and each individual. These GOFs were used to indicate the fit or degree to which each individual’s network was normative, with a higher GOF indicating a more normative network. These procedures yielded four GOF values for each individual (two pre-surgery and two post-surgery, one for each network). A repeated-measure ANOVA was run to test the effects of sessions (pre-/post-surgery for patients, or session one and two for the NCs) and experimental group (RTLE, LTLE, NCs) on the GOF, run separately for each network.

#### Regional differences

At the group level, statistical analyses were computed in order to determine spatial extent differences for each network between the groups and the sessions. For this, individual *Z*-maps were entered into a second-level random-effects analyses, for each network separately. The first analysis was done on the pre-surgery data only. In other words, we tested the differences between the three experimental groups, pre-surgery, for each DMN subdivision independently. Second, to analyze within-subject pre- to post-surgery changes, a difference image was created for each participant. This image was obtained by subtracting pre- to post-surgery *Z*-maps, for each subject and each network. This allowed us to test both for decreases (pre > post-surgery) and increases (post > pre-surgery) in spatial extent across the scanning sessions. As the number of days between the two scans was significantly different between the groups (see Results), we added this as a continuous variable, a covariate of no-interest, in the model. Each comparison was restricted to changes involving positive voxels losing or gaining their engagement in the network of interest. In other words, we did not report the clusters of voxels remaining below the threshold defining during the ICA (posterior probability threshold of *p* = 0.5; corresponding to a *Z* = 1.9 and 2.2 for the dorsal and vDMN, respectively) at both sessions despite a possible significant change between the two sessions, as any such pattern would indicate the regions were not part of the network of interest at either session.

In order to avoid any confounding effect between normal and true post-surgery changes, we utilized two independent additional analyses. First, at the whole-brain level, we recomputed the relevant patient contrast (i.e., post- versus pre-surgery), and applied an exclusive mask involving the regions associated with significant changes between the two sessions for the control group (*p* < 0.001, uncorrected). Second, for each cluster showing a significant change in either patient group, we computed the averaged *Z*-values in the control group and computed a paired *t*-test between the two sessions. Any clusters significant in the control group were excluded, as these would represent normative, not patient related, changes, and, therefore, are not presented in the Section “Results.”

Lastly, the height threshold of the statistical analyses was fixed at *p* < 0.001 (uncorrected, *T* > 3.31) and the spatial extent threshold at 50 voxels minimum for a cluster (e.g., corresponding to a corrected alpha level of *p* < 0.045).

#### Correlation with clinical characteristics

Lastly, we computed Pearson correlations to test the relation between the age of seizure onset and the GOFs, within each patient group. Also, regarding the analysis of regional differences, age of seizure onset was added as a covariate in the second-level analyses in order to test potential effects on pre-surgery DMN activity.

## Results

### Behavioral data

The three experimental groups did not differ by age (*p* = 0.7), nor gender (*p* = 0.09). With regard to the patient groups, the RTLE and LTLE groups did not differ by age of seizure onset, illness duration, number of anti-epileptic drugs (pre- or post-surgery), presence/absence of unilateral mesial temporal sclerosis (MTS) (pre-surgery), nor the time interval between the fMRI scans and surgery (Table 1). With regards to the specialization of the LH for language, the left TLE patients had a smaller resection than the right TLE patients. However, this difference was not significantly different between the patient groups. Regarding the seizure outcome of the patients, we used a classification based on the Engel classes [class I–IV; (25)], with an additional class V reflecting the report of post-operative pseudo-seizures. Overall, all of the LTLE patients were seizure free (Engel class I, at least 1 year after surgery), except one who was in class V at 1 year (e.g., reporting pseudo-seizures with no epileptic seizures). For the RTLE group, 14 of 16 patients also had a good seizure outcome [classes I (*N* = 10), II (*N* = 3), or III (*N* = 1)], with two patients in class IV with no change in seizure frequency (see Table 1).

The only difference that emerged between the groups involved the number of days between the two scans (*p* = 0.02). In detail, the time interval between scans was shorter for the control group (*m* = 380 days), than for the LTLE (*m* = 786 days; *p* = 0.02), but not for the RTLE group (*m* = 609 days, *p* = 0.3). Note that the TLE groups did not differ (*p* = 0.6). To correct for this difference, the time interval between scans variable was added as a covariate of no interest in the second-level analysis involving pre- versus post-surgery comparisons.

### Goodness-of-fit analyses

**Figure 2:**
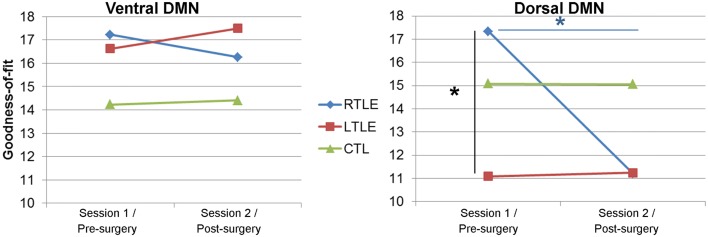
**Goodness-of-fit (GOF) for each default-mode network**. Left panel: dorsal DMN (dDMN), right panel: ventral DMN (vDMN). *Significant difference at *p* < 0.05.

A repeated-measure ANOVA applied on the GOF revealed no significant differences for the vDMN between the experimental groups (*p* = 0.4) or sessions (*p* = 0.3) (Figure 2). In contrast, for the dDMN, the RTLE showed higher GOF values than the LTLE, pre-surgery (*p* = 0.013). This difference between left and right TLE disappeared post-surgery, as we observed a significant reduction of the RTLE’s post-surgery GOF (*p* = 0.013), reaching a level almost identical to that of the LTLE group. This effect was mostly driven by the high pre-surgery GOF of the RTLE, as it did not remain significant when accounting for this parameter in the model (by adding the pre-surgery GOF values as a baseline covariate). Of note, neither of the TLE patient groups’ GOF significantly differed from the controls’, for either network, pre- or post-surgery.

Within the patient groups, a negative correlation was revealed between the age of seizure onset and the GOF for the pre-surgery dDMN (*r* = −0.42; *p* = 0.02), indicating that more normative dDMN was associated with earlier age of seizure onset. Both TLE groups had similar effects (LTLE: *r* = −0.45, RTLE: *r* = −0.47). No other significant correlations were observed between age of seizure onset and the GOF values for either sub-network at either the pre- or post-surgery testing point.

### Regional analyses

#### Pre-surgery

Consistent with the GOF analysis, the dDMN showed more regional differences between the three experimental groups than the vDMN (Table 2). In detail, both networks showed significant differences between the experimental groups, or more specifically larger differences between the right and left TLE, than between the patient groups (RTLE and LTLE separately) and NCs.

**Table 2:** **Description of the significant differences within each DMN subdivision, between the experimental groups, pre-surgery**.

Contrast	Region	Cluster voxel	*Z*-value	*x*	*y*	*z*	*Z* (RTLE)^a^	*Z* (LTLE)^a^
**DORSAL DMN**								
RTLE–LTLE	R Sup Ft	167	5.03	20	52	10	17	4
	R Sup Ft		3.71	20	56	22		
	R Sup Ft	139	4.25	14	26	−16	27	12
	R Rectus		4.22	8	44	−14		
	R Sup Ft	194	4.18	12	44	48	22	4
	R Sup Ft		3.84	12	28	44		
	R Sup Ft		3.81	24	38	48		
	L PCC	59	4.05	−4	−38	30	10	−1
	L PCC		3.45	−10	−44	26		
LTLE–RTLE	Null							

							*Z* (TLE)^a^	*Z* (NC)^a^

NC–LTLE	Null							
NC–RTLE	R Sup Tp	58	4.27	58	6	4	−4	9
LTLE–NC	L Cereb	63	4.11	−48	−76	−30	3	−11
	L Cereb		3.39	−42	−88	−32		
RTLE–NC	Null							
**VENTRAL DMN**								
RTLE–LTLE	L PCL	125	5	−10	−30	68	6	−2
	L Precu		3.47	−8	−44	68		
	R Mid Tp	124	4.02	52	−40	4	14	1
	R Mid Tp		3.28	60	−52	0		
LTLE–RTLE	L Cereb	92	3.99	−12	−72	−34	−3	7
	Cereb		3.42	0	−72	−38		

							*Z* (TLE)^a^	*Z* (NC)^a^

NC–LTLE	L Cereb	68	4.19	−16	−50	−18	−9	5
NC–RTLE	Null							
LTLE–NC	Null							
RTLE–NC	R Calcar	51	4.12	28	−66	12	3	−4
	L Precu	63	3.87	−6	−42	68	26	9
	L Precu		3.69	−2	−50	64		

*NC, normal controls; LTLE, left temporal lobe epilepsy; RTLE, right temporal lobe epilepsy; Cereb, cerebellum; Calcar, calcarine; Precu, precuneus; Tp, temporal cortex; Mid, middle; L, left; R, right; PCL, paracentral lobule; Sup, superior; Ft, frontal cortex; PCC, posterior cingulate cortex*.

*^a^ The last two columns indicate the averaged *Z*-values within each cluster for each group of interest, for each contrast, indicating the degree of difference between the two groups*.

**Figure 3:**
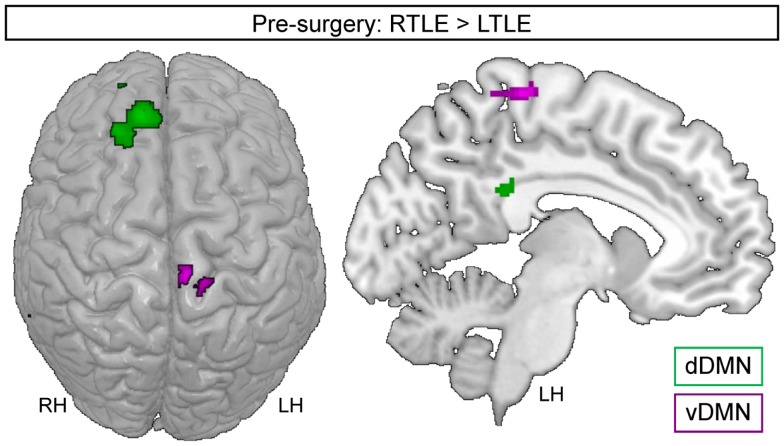
**Regions showing increased engagement in RTLE, relative to LTLE group, for the dorsal DMN (green) or the ventral DMN (purple)**. The images are shown in radiological orientation [e.g., the right side is the left hemisphere (LH)]. LH, left hemisphere; RH, right hemisphere.

Regarding the dDMN, the largest differences in pre-surgery emerged between the TLE groups. Indeed, the RTLE patients showed increased engagement of three large clusters located in the right superior frontal cortex and one cluster in the left PCC (Figure 3). In contrast, the LTLE did not display regions with significantly increased engagement in the dDMN, relative to the RTLE group. Compared to the NCs, the RTLE group showed reduced engagement of the right superior temporal cortex, while the LTLE group had an increased engagement of the left cerebellum (Crus I) in this DM subnetwork.

Regarding the vDMN, the RTLE group also showed increased engagement of large clusters, compared to the LTLE patients (Table 2). They were located in the left paracentral lobe, extending to the precuneus (Figure 3), and in the right middle temporal cortex. In contrast, the LTLE group showed increased involvement in the VIIB lobule of the left cerebellum, relative to the RTLE group. But the LTLE also displayed reduced involvement in the lobule V of the left-sided cerebellum, relative to the control group. Finally, we revealed that the RTLE group demonstrated increased engagement in the right calcarine and the left precuneus but no significant reduction compared to controls.

No association was revealed between the age of seizure onset and either pre-surgery DMN within each patient group.

#### Change from pre- to post-surgery

**Figure 4:**
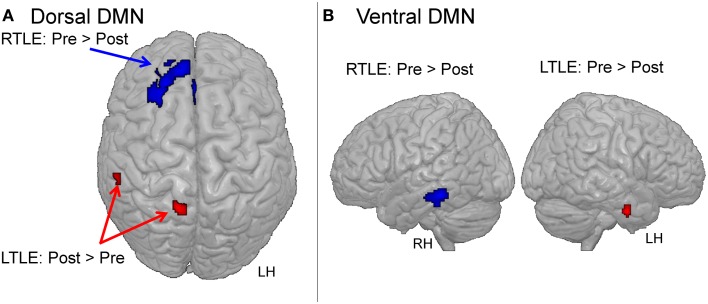
**Major changes between pre- and post-surgery for the dorsal DMN (A) or the ventral DMN (B), within each patient group**. The images are shown in radiological orientation (e.g., the right side is the left hemisphere). LH, left hemisphere; RH, right hemisphere.

Overall, comparisons between the pre- and post-surgery sessions demonstrated larger changes in the dDMN than in the vDMN for both patient groups (Table 3). For the dDMN, the LTLE demonstrated increased involvement in several regions outside the ictal temporal lobe, post-surgery in comparison to pre-surgery. In detail, the right precuneus, right inferior parietal, and left middle temporal (posterior to the resection area) clusters showed positive involvement in the dDMN, post-surgery relative to pre-surgery (Figure 4). In contrast, as expected, the LTLE group lost the engagement of the left parahippocampal gyrus, located in the resected area, but also a cluster in the right middle temporal gyrus, post-surgery. The RTLE patients did not show any significant increased involvement of other regions in the dDMN, post-surgery. On the contrary, there was greatly reduced involvement of ipsilateral regions in their dDMN. Most notably, a particularly large focal cluster located in the right frontal lobe showed lower participation in the dDMN activity (Figure 4). To a lesser degree, the right caudate nucleus and insula also lost their functional involvement in this network post-surgery. Importantly, none of these changes were significant in the control group. NCs only showed a small reduction of engagement of the right thalamus in the dDMN during the session 2, relative to the session 1. We did not observe any significant increase at session 2 for the controls.

**Table 3:** **Description of the changes between the two sessions, within each experimental group, for each DMN subdivision**.

Contrast	Region	Cluster voxel	*Z*-value	*x*	*y*	*z*	*Z* (pre)^a^	*Z* (post)^a^
**DORSAL DMN**								
LTLE: post > pre	R Precu	138	4.68	16	−54	66	−9	3
	R Inf Pt	50	4.43	66	−30	52	0	7
	L Mid Tp	65	4.09	−52	−34	−4	−2	7
LTLE: pre > post	L Parahip – resected area	123	4.27	−28	−22	−22	2	N/A
	R Mid Tp	98	3.89	60	−60	4	4	− 6
RTLE: post > pre	Null							
RTLE: pre > post	R Sup Ft	690	4.49	12	46	50	19	4
	R Sup Ft		4.34	22	36	48		
	R Sup Ft		4.18	26	26	50		
	R Caudate	69	3.59	14	16	16	9	− 1
	R Insula	119	3.82	40	16	−14	22	3
NC: ses 2 > ses 1	Null							
NC: ses 1 > ses 2	R Thalamus	61	3.9	2	−6	10	6	− 2
**VENTRAL DMN**								
LTLE: post > pre	Null							
LTLE: pre > post	L Inf Tp – resected area	61	4.31	−56	−2	−32	13	N/A
RTLE: post > pre	Null							
RTLE: pre > post	R inf Tp – resected area	294	5.05	66	−34	−16	9	N/A
	R inf Tp – resected area		4.23	60	−30	−26		
NC: ses 2 > ses 1	Null							
NC: ses 1 > ses 2	L Precu	143	4.66	−14	−54	26	24	19
	L Mid Tp	66	3.89	70	−34	−4	7	1

*NC, normal controls; LTLE, left temporal lobe epilepsy; RTLE, right temporal lobe epilepsy; Cereb, cerebellum; Precu, precuneus; Tp, temporal cortex; Mid, middle; L, left; R, right; Inf, inferior; Sup, superior; Ft, frontal cortex; Parahip, parahippocampal gyrus; Pt, parietal cortex*.

*^a^ The last two columns indicate the averaged *Z*-values within each cluster for each session (pre- and post-surgery for the patients, sessions 1 and 2 for the controls), indicating the degree of change between the sessions within the experimental group*.

Regarding the vDMN, the only changes evident post-surgery were in the ictal/resected temporal lobe for each patient group, with reduced engagement, as expected (see Table 3). Also, the NCs showed reduced activity in two clusters located in the left precuneus and left middle temporal lobe for the session 2, relative to the session 1.

## Discussion

The present study investigated differences in the spatial extent of the two major subdivisions of the well-known DMN at rest in TLE patients. We tested for differences before and after ATL, with close examination of side of pathology (i.e., ictal focus) as a mediating factor. Prior work has described aberrant activity in the overall DMN in TLE patients’ pre-surgery (11). We go further by clearly showing the unique effects of left versus right TLE, with further demonstration of the effects of ATL on the two main subdivisions of the DMN. Indeed, in contrast to our initial hypothesis, we found more evidence of TLE group differences in the dorsal rather than the vDMN, including distinct patterns of change for dDMN post-surgery. Thus, we show that ATL does not affect the DM networks in an equivalent fashion, and the nature of its impact varies depending on the pathologic hemisphere involved. Our results, therefore, imply that ATL has distinct effects on the cognitive functions associated with the DMN subdivisions, and that these effects will vary as a function of right and left TLE.

Importantly, our data show little evidence that the ventral subdivision of the DMN was affected by either TLE pre-surgery or ATL. Indeed, at the whole-brain level, using GOF measures, our analyses failed to produce significant differences, while we did observe substantive differences in the dDMN between the groups and the sessions. This seems counter-intuitive as the vDMN includes the epileptogenic mesial temporal lobe in TLE. However, within the vDMN, the mesial temporal lobe has a much less pivotal role than the precuneus, a region considered to be a major hub in the DMN (26). Therefore, the relative integrity of this network may suggest that the precuneus plays a compensatory role, reducing the negative impact of mesial temporal disease over the rest of the network, and otherwise helping to maintain network integrity. As a matter of fact, our regional analyses revealed little differences between the patient and control groups, supporting this interpretation. Further emphasizing a compensatory role for the precuneus in our data is that the RTLE group showed more engagement of the precuneus in the vDMN, than either the LTLE or controls. This finding is consistent with Zhang et al.’s study (11), which suggested that the PCC may play a compensatory role for the altered DMN in right but not left TLE. Our findings in LTLE for the vDMN stand in stark contrast to this, compelling consideration of an alternative interpretation. For example, rather than proposing that the RTLE is displaying a compensatory response involving the precuneus/PCC area (suggesting that the LTLE is associated with a normal activity in this region), instead the LTLE patients can be seen as showing a pathologic loss of engagement of this region in the vDMN.

It is also worth noting that the LTLE group showed distinctly different engagement of the left-sided cerebellum with the rest of the vDMN, relative to the two other experimental groups. The exact reason for this cortical-cerebellar alteration is unclear, pre-surgery. It is interesting to note that in our previous study we observed a specific modulation of the functional connectivity between the left hippocampus and the left-sided cerebellum during a working memory task in the LTLE but not the RTLE (27). Thus, in combination, these separate results for LTLE patients may represent accruing evidence for altered functional connectivity between the cortex and the left-sided cerebellum. Overall, the absence of large alterations within the vDMN indicates that the abnormalities of unilateral temporal lobe seizures do not cause major perturbation of the whole vDMN. As was the case with the right TLE group, we again suggest that the precuneus/PCC has a protective role within this mostly posterior network, preventing major changes that might otherwise be caused by TLE pathology. This role is supported by previous studies describing the precuneus/PCC as a principal hub in the DMN (26, 28), perhaps generating activity or signals that limit seizure spread or epileptogenesis in TLE patients (29).

In contrast, our data show that TLE and ATL do have a significant effect on the regional extent of the dDMN, a sub-network whose major hub is prefrontal cortex, with precuneus involvement present, but to a much smaller degree. Indeed, large discrepancies were evident as a function of the side of the pathology. Pre-surgery, based on the GOF, the RTLE showed a more normative network than the LTLE (i.e., higher GOF as a sign of a better matching to the template). Also, the present data demonstrate that, pre-surgery, the LTLE group showed reduced involvement of the major regions in this network such as frontal cortex and PCC, relative to the RTLE group. Differences between the impact of right and left TLE have been described previously, associated with the (dominant) function of the LH in language-related processes (14, 15). It is important to note that the majority of our participants were right-handed, suggesting LH dominance for language was prominent in our sample. Thus, our data support the notion that the functions of the dDMN may be more strongly implemented by the LH, and is consistent with previous finding suggesting that the DMN is left-lateralized, heavily involved, for instance, in functions such as inner, covert speech (30). Given this lateralized bias in function, it makes sense that left-sided seizures are more harmful to this network than right-sided seizures. Conversely, our data suggest that the vDMN is less engaged in language processing, and will be less sensitive to left-lateralized pathology such as LTLE.

Another striking result is the negative correlation revealed between the age of seizure onset and the dDMN’s GOF in patients, pre-surgery, regardless of the side of the pathology. In other words, this indicates that earlier age of seizure onset is associated with a more normative network. Indeed, higher GOF is indicative of a better match between the patients’ network and the “normative” dDMN obtained by Shirer et al. (24) on healthy participants. We failed to find evidence of an association with onset at the regional level, suggesting that age of seizure onset has more of a global influence on the whole network, rather than selectively impacting a specific network region. This negative correlation is consistent with literature addressing the influence of early versus late seizure onset on brain plasticity. Indeed, previous studies describe how a mature brain is less plastic, allowing late onset seizures to cause irreversible impairment in the setting of fully acquired and developed cognitive functions (31). Our result suggests that this developmental feature is at work with the dDMN, implying that when a young brain is confronted with seizures it is more capable than an older brain of generating functional adaptation and plasticity in the dDMN. We observed no such developmental relationship in the vDMN.

Our study is the first to investigate the effect of ATL on DMN subdivisions, and, in addition, reporting different effects according to the side of the ATL. It is important to remember that the majority of our patients were confirmed to have good seizure outcome (seizure free or significant reduction of their seizures) at least 1 year after their surgery, confirming that the ATL surgery likely resected the primary seizure onset zone. Our data show that right ATL causes more damage in the dDMN than left ATL. More specifically, right ATL, but not left ATL, was associated with a large reduction in engagement of ipsilateral regions, especially the right frontal cortex in the dDMN. The reason for such loss of activity is still not clear, and we are the first to report this finding. This loss of frontal activity in the RTLE patients implies that right frontal cognitive deficits are likely to be more prominent in these patients following ATL, though neuropsychological studies, involving measures of executive function, do not support this (32). One possibility for this discrepancy is that diminished right frontal dDMN activity may impact other types of cognitive processing not measured by standard neuropsychological testing such as spontaneous cognition (e.g., mind wandering). Indeed, DMN activity has been associated with spontaneous mental processes in healthy subjects (3, 4), but no study has explored nature and integrity of spontaneous thoughts in TLE patients with concurrent measures of DMN subdivision activity.

With regard to the unique effects of the side of the ATL, left but not right ATL patients showed recruitment of additional posterior regions into the dDMN post-surgery. This finding speaks to the differential effect of temporal lobe seizures on the dominant versus non-dominant hemisphere. It has been previously demonstrated that LTLE patients are more prone to brain activity abnormalities than RTLE, pre-surgery (14, 15). Yet, we observed an increased engagement of the precuneus in this network for the LTLE patients post-surgery relative to pre-surgery. This is consistent with our previous interpretation regarding the positive and possible compensatory effect of this region on the network as a whole. Recalling that our left TLE patients had good seizure outcomes, we suggest that, released from seizure burden, the left dominant hemisphere is able to function more normally, with this normalization supported through the compensatory benefits conferred by adding new regions to bolster dDMN activity. This idea is also convergent with the findings of McCormick et al. (10), who described that post-surgical enhancement of functional connectivity involving the PCC correlated with better post-surgical episodic memory performance.

Overall, our data indicate that right TLE is associated with greater disruption of the dDMN post-surgery. In terms of lateralized effects, our data differs from the most common reports in the neuropsychological literature, which generally indicate that left (i.e., dominant hemisphere) TLE patients fare worse than right TLE patients after ATL surgery (33, 34), particularly in areas such as episodic memory. While our data may imply left, not right, sided ATL engender adaptive mechanisms and compensatory network responses after surgery, we cannot presume that the post-surgical changes observed in our right TLE are either associated with cognitive outcome or could explain any observed neurocognitive findings. Further investigations are needed to fill the gap between our knowledge of functional reorganization and its consequences for cognitive change following ATL and other surgical interventions.

With regard to the vDMN and surgery, our data suggest that ATL had very limited effects. Indeed, we only found expected changes within the resected temporal lobe for each of the patient groups. This lack of change is consistent with our interpretation of the pre-surgical finding. That is, the engagement of the precuneus/PCC area may have worked to limit or constrain some of the disruptions in the network caused by resection of the epileptogenic temporal lobe. More generally, these findings for the vDMN imply that TLE and ATL surgery, regardless of side, has less of an impact on vDMN functionality (e.g., decision-making related to constructing/recalling a mental scene) than might be expected.

Some limitations in our study should be noted. We cannot exclude the possibility that some changes revealed between pre- and post-surgery in the patient groups were partially caused by normal aging or the normal effects of time. Related to this, we were not able to match exactly the NC and the LTLE participants on the time interval between the scans. As described, we checked that the brain regions displaying change between the two sessions for the TLE patients were not areas of change in the NC group. Furthermore, we did not find any regions with increased engagement in the control group, suggesting that such DMN alterations are quite specific to the effects of ATL surgery and are not likely related to normal, time-related change in the healthy participants. Thus, we believe that we have highlighted the reliable effects of the ATL procedure on DMN activity at rest in TLE patients.

Another limitation is that the patients were not all seizure free. The right TLE group had slightly fewer seizure-free patients than the left TLE group, post-surgery (11/16 versus 12/13), although this difference was not significant. Unfortunately, the low sample size of the non-seizure free group precluded statistical analyses of the potential effect of different seizure outcomes on our findings. We acknowledge that patterns of seizure recurrence versus control may influence the status of the DMN. Indeed, it could be playing a role in our finding that the right, but not the left, TLE patients suffer from a large reduction of frontal activity in the dDMN as a result of factors such as seizure spread or secondary epileptogenesis [see Ref. (35)]. Further investigation is needed to explain such phenomenon, using a more balance sample size of TLE patients with poor versus good seizure outcome.

Regarding other clinical factors, AEDs could potentially effect network connectivity and changes post-surgery. However, AED status did not vary or change after surgery in our sample. We considered analyzing collected clinical data on seizure frequency. However, the experience at our epilepsy center is that age of onset is a more reliable measure than other historical measures such as seizure frequency or age at first risk (e.g., first signs of pathology). The former because awareness and recall of seizure occurrence is so poor; the latter because of the potential delay between the start and the discovery of pathology. We chose to focus on age of seizure onset as this may best capture developmental differences in response to neuroplasticity. For instance, younger compared to older age brains appear more disposed to plasticity and cognitive reorganization (36), factors that likely play a role in DMN strength and organization. With these issues of neuroplasticity in mind, variables such as – the age at which seizures fail to respond to medications – would not be as accurate, nor as meaningful. Moreover, our algorithm for ATL candidacy requires that all patients fail at least three seizure medications, with a large number having many more such failed trials. Therefore, it would be very difficult to accurately determine the age at which seizures failed to respond to medication. Lastly, illness duration is highly correlated with age at seizure onset, and thus constitutes a redundant variable.

Regarding pathology, it should be noted that all our patients did not have the same temporal pathology, especially with regard to the presence or absence of MTS. While we are aware that this variable is an important and relevant factor in TLE (37), our sample size was too low for any meaningful statistical comparison between patients with and without MTS. It should be noted that our major purpose was to explore the extra-temporal effects of TLE on network connectivity, and these extra-temporal regions were not “lesioned” in any of our patients.

## Conclusion

We demonstrate that the subdivisions of the well-known default-mode resting-state network are effected differently by both the original TLE pathology, and subsequent ATL procedure. Overall, and somewhat unexpectedly, the dDMN appears more impacted by these factors. Prior to surgery, major whole-brain differences were observed in the dDMN, with right TLE displaying a more normative pattern based on the GOF measures. In terms of regional dDMN effects, the TLE group differences observed in frontal and precuneal regions, imply that left-sided, dominant hemisphere, pathology is more damaging to the network. In contrast, the status of the vDMN prior to surgery showed no differences among our groups at a whole-brain level, suggesting that neither left nor right pathology has a detrimental effect. Regionally for the vDMN, the RTLE group demonstrated increases, particularly in the precuneus, increases we believe reflect an adaptive, neuroprotective response, compensating for the right mesial pathology. Regarding the impact of ATL and post-surgical change in the DMN subdivisions, the vDMN, which includes most of the mesial temporal lobe, did not demonstrate any significant loss of activity outside the resected epileptogenic cortex, a finding that again suggests that the vDMN is less affected by epileptogenic pathology. For the dDMN post-surgery, contrasting effects were obtained for the TLE groups. LTLE patients demonstrated increased engagement of new posterior regions such as the precuneus, while the RTLE patients showed lost engagement of a large right anterior cluster. Here, we again see an adaptive or compensatory role for the precuneus, though in this case in the setting of left TLE.

Overall our data demonstrate that right ATL has a more deleterious effect on the dDMN, and that left, not right, sided ATL appears more likely to engender adaptive mechanisms and compensatory network responses after surgery. This latter suggests a possible inconsistency with the neuropsychological data, which tend to associate dominant hemisphere ATL with greater functional problems post-surgery in domains such as episodic memory. In this sense, our data raise questions about the nature and extent of the correspondence between resting-state networks associated with memory (i.e., the DMN) and neuropsychological measures of functionality. Further investigations are needed to fill the gap between knowledge about functional reorganization, as reported here, and its impact on cognitive status post-surgery.

To our knowledge, we are the first to highlight the differential impact of right and left TLE and subsequent ATL on the major subdivisions of the DMN, at rest. Our data show that the dDMN is more closely associated with the impact of TLE pathology and resective surgery than the vDMN, suggesting that studying the cognitive and behavioral correlates of this DMN subdivision and its changes with surgery may be fruitful, particularly as resting-state becomes better integrated into pre-surgical algorithms for predicting neurocognitive, neurobehavioral, and seizure outcomes.

## Author Contributions

Gaëlle E. Doucet conducted, performed, analyzed imaging experiments, and wrote the manuscript. Dorian Pustina performed imaging experiments. Christopher Skidmore and Michael R. Sperling designed and supervised the project. James Evans and Ashwini Sharan performed ATL surgeries. Joseph I. Tracy designed, supervised the project, and wrote the manuscript.

## Conflict of Interest Statement

The authors declare that the research was conducted in the absence of any commercial or financial relationships that could be construed as a potential conflict of interest.

## Footnotes

^1^http://www.fil.ion.ucl.ac.uk/spm/software/spm8

^2^http://fsl.fmrib.ox.ac.uk/fsl/fslwiki/
